# Circadian Rhythms of Fetal Liver Transcription Persist in the Absence of Canonical Circadian Clock Gene Expression Rhythms *In Vivo*

**DOI:** 10.1371/journal.pone.0030781

**Published:** 2012-02-23

**Authors:** Chengwei Li, Shuang Yu, Xiaoling Zhong, Jianguo Wu, Xiaodong Li

**Affiliations:** National Key Laboratory of Virology, College of Life Sciences, Wuhan University, Wuhan, Hubei Province, People's Republic of China; Vanderbilt University, United States of America

## Abstract

The cellular circadian clock and systemic cues drive rhythmicity in the transcriptome of adult peripheral tissues. However, the oscillating status of the circadian clocks in fetal tissues, and their response to maternal cues, are less clear. Most clock genes do not cycle in fetal livers from mice and rats, although tissue level rhythms rapidly emerge when fetal mouse liver explants are cultured *in vitro*. Thus, in the fetal mouse liver, the circadian clock does not oscillate at the cellular level (but is induced to oscillate in culture). To gain a comprehensive overview of the clock status in the fetal liver during late gestation, we performed microarray analyses on fetal liver tissues. In the fetal liver we did not observe circadian rhythms of clock gene expression or many other transcripts known to be rhythmically expressed in the adult liver. Nevertheless, JTK_CYCLE analysis identified some transcripts in the fetal liver that were rhythmically expressed, albeit at low amplitudes. Upon data filtering by coefficient of variation, the expression levels for transcripts related to pancreatic exocrine enzymes and zymogen secretion were found to undergo synchronized daily fluctuations at high amplitudes. These results suggest that maternal cues influence the fetal liver, despite the fact that we did not detect circadian rhythms of canonical clock gene expression in the fetal liver. These results raise important questions on the role of the circadian clock, or lack thereof, during ontogeny.

## Introduction

Endogenous circadian clocks are of adaptive value for organisms to cope with daily environmental change [1]. Circadian rhythms exist at the cellular level [2], [3], whereby expression of clock genes form transcriptional feedback loops that are at the core of cellular oscillations [4], [5]. In mammals, the central clock in the suprachiasmatic nucleus (SCN) of the hypothalamus can respond to light-based information from the environment and adjust its phase accordingly [6]. In peripheral tissues, the circadian clocks orchestrate a variety of cellular functions [7], [8]. Rhythmic transcripts, on the other hand, may have distinctive expression profiles between different tissues that reflect specialized functions [7], [8]. In the liver, only a small subset of genes are directly under clock control, while feeding and systemic cues have significant influence on rhythmicities within the liver transcriptome [9], [10]. Peripheral clocks in different tissues have distinct phase relationships with the central clock, forming a hierarchical organization of the circadian timing system [11], [12]. Disruption of this internal harmony is detrimental to health [5], [13].

Compared to the extensive research that has assessed the roles of the circadian clock in adult physiological processes, the functions of circadian oscillations during embryonic and fetal development have not been as well studied and remain to be fully elucidated. Early studies on the ontogeny of clock functions indicated that the SCN clock in rodents began oscillating before birth [14] and could be entrained by exogenous agents and/or maternal rhythms [15], [16]. However, recent studies on developmental expression of clock genes indicate that the prenatal molecular oscillations are typically weak [17], [18]. The more robust oscillations of the SCN manifested rather quickly during postnatal development [17], [18]. In peripheral tissues, analyses of clock genes' developmental expression profiles indicate that adult-like patterns were not established in the rat liver until the weaning period [19], and postnatal appearance of circadian oscillation varies among different tissues [20]. Studies on fetal tissues during late gestation in mice and rats failed to detect any rhythmic expression at the tissue level for clock genes [19], [21]. Intriguingly, robust circadian oscillations could be detected at the tissue level in mouse fetal liver explants after about one day in culture [21]. This raised the possibility that fetal liver cells possessed oscillating clocks but were not synchronized *in utero* by maternal rhythms. However, mouse embryonic fibroblasts (MEFs), upon transplantation into adult mice, could be readily entrained by the host [22]. It is uncertain why maternal cues did not appear to do so to the fetal tissues. One possibility is that fetal tissues are in a constant *in utero* milieu that is shielded from daily variations of systemic cues from the dam. A more likely possibility is that maternal cues did in fact influence the fetal cells, but clock genes in the fetal cells fail to respond. Such abnormalities in clock gene expression could also account for lack of circadian oscillations at the cellular and tissue levels.

To gain insights into the clock's oscillation status and the potential influence of maternal cues on the fetal liver transcriptome, we performed microarray analysis on fetal liver tissues during late gestation. We did not detect circadian rhythms in transcript abundance for nearly all of the clock genes and many of the established clock-controlled genes. However, some transcripts were found to be rhythmically expressed or exhibit daily fluctuations in the fetal liver, possibly in response to maternal cues. The results suggested that expressions of clock genes were regulated by distinct molecular mechanisms during late gestation in the fetal mouse liver.

## Materials and Methods

### Liver tissue collection

This study was carried out in accordance with the recommendations in the Guide for the Care and Use of Laboratory Animals from the National Institutes of Health. The study protocol was approved by the Committee on Experimental Animals of the Science and Technology Department of Hubei Province, China (Permit Number: SYXK 2006-0037). Adult C57BL/6 mice were obtained from the Experimental Animal Center of Wuhan University and housed under LD cycle (12 hrs light/12 hrs dark) conditions with unrestricted access to food and water. Female and male mice were paired together during the light period and left overnight. The male mouse was then removed from the cage after lights-on in the next day. After mating, the female mice were housed under the same LD cycle. Before fetal (E18 and E19) tissue collection, the pregnant mice were placed under DD conditions (see Figure S7 for tissue collection schedule). For each time point, two or three pregnant mice were sacrificed and the fetal livers (10–15 in total) were minced and pooled together as one sample for analysis. Livers were also collected from adult male mice (about 3 months old) at every four hours over a single day (3 mice per time point), using the same schedule as for fetal tissue collection.

Two batches of female mice were mated. In the first batch (series 1), male mice were introduced during the beginning of the light cycle and subsequently left with the female for approximately 24 hrs. Some successful mating occurred during the light phase, immediately after pairing, while others occurred later. It was expected that there should be more variations in actual developmental timings of the fetuses. We used fetal tissues from this batch of mating as a control and replicate. In the second batch (series 2), male mice were introduced just before the end of the light cycle, during the light period, and were left overnight before separation in the next morning (∼13 hours). All dissected tissues were immediately stored in liquid nitrogen until required for processing to extract RNA.

### RNA analysis

Frozen tissues were ground in liquid nitrogen with Trizol and left in Trizol. Aliquots of each of the Trizol/tissue suspensions were sent out for commercial microarray hybridizations at CapitalBio (http://www.capitalbio.com/). The remaining suspension was stored at −80°C for subsequent on-site RNA isolation. Integrity of the isolated RNA was checked by agarose gel electrophoresis and RNA concentrations were measured with RNA standards using the Qubit fluorometer (Invitrogen, USA). A total of 1 ug of RNA isolated from each embryonic or adult time point was reverse transcribed. For semi-quantitative RT-PCR, equal efficiencies of different reverse transcriptions were validated by PCR amplification of *Actb* for 20 or 23 cycles. Semi-quantitative RT-PCRs on other transcripts were performed for 23, 25, 27 or 30 cycles according to transcript abundance and to allow clear contrast. Results presented are representative of duplicate or triplicate repeats. Controls were run using RNA templates but with no reverse transcription enzymes. Semi-quantitative RT-PCR products were visualized by agarose gel electrophoreses. The GenBank accession numbers for the genes and the PCR primers used in this study are listed in Table S6.

### Microarray hybridizations and data analyses

The GEO repository accession number for the fetal mouse liver microarray data presented in this study is GSE28622, which contained 24 sample files. We refer to GSM709400-GSM709411 as our series 1, and GSM709521-709532 as our series 2 throughout the text. RNA isolation, integrity validation, *in vitro* transcription (IVT) labeling and array hybridization and scanning were performed at CapitalBio (http://www.capitalbio.com/) using standard procedures. For each series, 12 Affymetrix mouse genome 430A 2.0 chips were processed in parallel. In total, 24 chips were used to assess the two separate series. Hybridized chips were scanned and the acquired raw data files (.CEL) were returned to the authors for further data analysis. All data are MIAME compliant. The robust multi-array average with GC-content background correction (GC-RMA) probe summarization algorithm was used for data normalization (natural or log2 scale). The two microarray data series were processed separately. JTK_CYCLE analyses were performed on normalized expression values (excluding probe sets for Affymetrix control genes. Complete data series of 48 h were used) to detect rhythmic gene expressions. Coefficients of variations and maximum/minimum values across time points within each time series were calculated for all probe sets to facilitate data analysis. Raw data files for GSE11923, GSE13093 and E-MEXP-842 were downloaded from the GEO and ArrayExpress and similarly analyzed. Expression values obtained by our calculations differed slightly from those posted on the websites, possibly due to differences in software versions used and/or differences in samples' groupings for parallel GC-RMA processing. This did not affect our conclusions. For consistency, circadian period ranges were specified as between 20 to 28 hours for JTK_CYCLE analyses of all data series. GC-RMA and JTK_CYCLE analyses were performed using the R statistical software package (version 1.12.1). The Heatmap Builder program was used to generate heatmaps corresponding to gene expression values detected.

## Results

### Fetal liver lacks detectable circadian rhythms of clock gene expression

**Figure 1:**
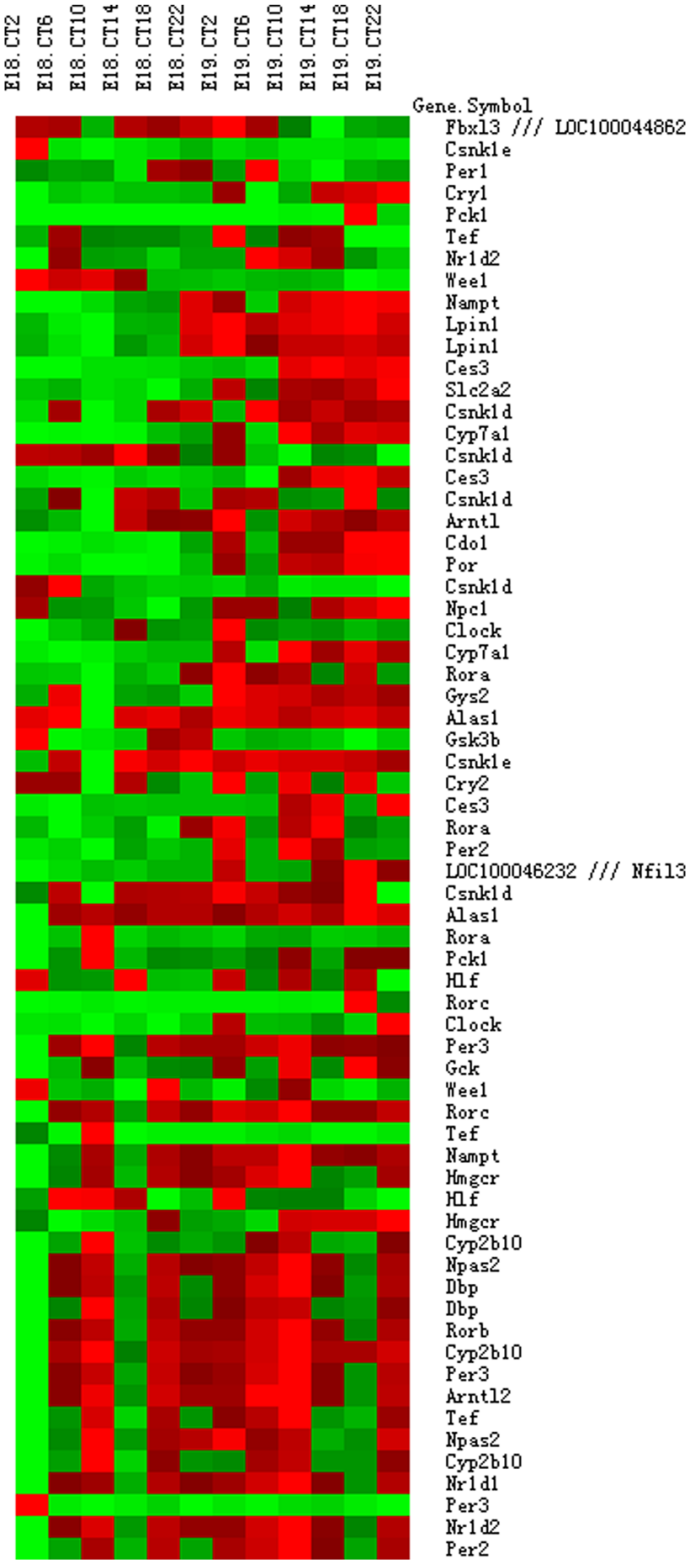
Heatmap of 66 probe sets representing clock and known rhythmic genes. Natural scale expression values from series 2 data for core clock genes and genes known to be rhythmically expressed in the adult liver were plotted with Heatmap builder. Some genes were represented by multiple probe sets. Phases were sorted by the “lag” values given by JTK_CYCLE.

A previous quantitative RT-PCR study of fetal mouse liver had failed to detect circadian rhythms of clock gene expression [21]. Consistent with this, our JTK_CYCLE [23] analyses of GC-RMA [24] normalized fetal liver expression values on embryonic day (E)18 and E19 indicated that nearly all the known clock genes did not show circadian rhythms of expression in the fetal liver, including *BMAL1/Arntl*, *BMAL2/Arntl2*, *CLOCK*, *Cry1*, *Cry2*, *Csnk1d*, *Csnk1e*, *Fbxl3*, *Npas2*, *Per1*, *Per2*, *Per3*, *Rora*, *Rorb*, *Rorc*, *Rev-erb α*/*NR1D1*, *and Rev-erb β*/*NR1D2* (Table S1). Many genes known to be rhythmically expressed in the adult mouse liver also did not show circadian rhythms of expression, such as *Alas1* (Delta-aminolevulinate synthase 1), *Ces3* (carboxylesterase 3), *Cyp7a1* (cytochrome P450 7a-hydroxylase), *Cyp2b10*, *Dbp*, *Gck* (glucose kinase), *Slc2a2/GluT2*, *Gys2* (glycogen synthase 2), *Hlf* (hepatic leukemia factor), *Lpin1* (lipin-1), *Nampt* (nicotinamide phosphoribosyltransferase), *Pck1* (phosphoenolpyruvate carboxykinase 1), *Por* (cytochrome P450 oxidoreducatase), *Tef* (thyrotroph embryonic factor) and *Wee1* [7], [25], [26], [27], [28]. Overall, we evaluated 66 probe sets representing clock genes and known rhythmic transcripts in the adult liver (Figures 1 and S1; Table S1). Few rhythmic transcripts were identified in either of our two data series by JTK_CYCLE analysis, and none were common to both data series. Furthermore, most transcripts showed peak to trough variations of less than 2.5 fold over the two days of late gestation. Taken together, our results strengthened the view that circadian regulatory cycles are not present in the fetal mouse liver at the tissue level.

### Detection of rhythmic transcripts at low amplitudes by JTK_CYCLE

**Figure 2:**
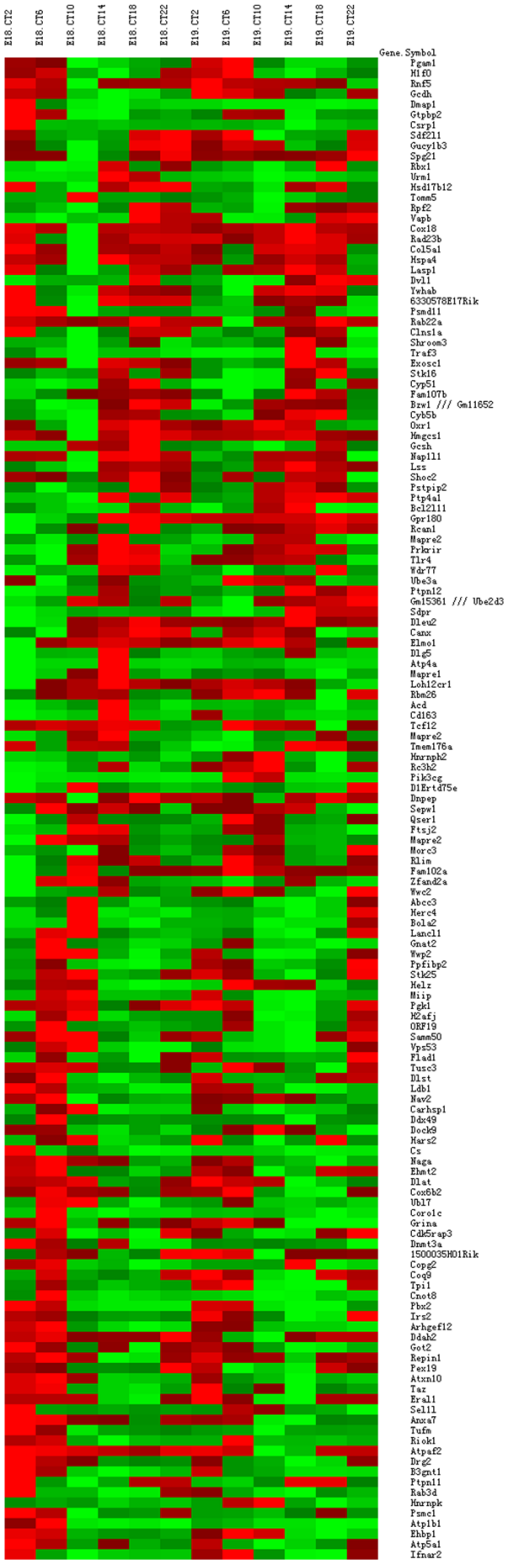
Heatmap of the 145 rhythmic probe sets in series 2 microarray data. Series 2 natural scale expression values for the 145 probe sets that were rhythmic in both series of data (*p*<0.1 by JTK_CYCLE) were plotted. Phases were sorted by the “lag” values given by JTK_CYCLE.

Despite the failure to detect circadian rhythms in fetal liver for the set of transcripts known to have robust expression rhythms in the adult liver, JTK_CYCLE analysis revealed many rhythmic transcripts (criteria: *p*<0.1) in each of our data series. However, the *p*-values after corrections for multiple measurements were rather high (*BH*.*Q* values>0.4), suggesting that these genes were less likely to be truly rhythmic. Furthermore, the amplitudes of the expression rhythms were generally low for those rhythmic transcripts, with most probe sets showing less than 2.5-fold changes between expression peaks and troughs for the 12 time points examined. Nonetheless, a common set of 145 probe sets was found to be rhythmic in both series of data (Figures 2 and S2; Table S2). DAVID analyses [29] on those common rhythmic transcripts revealed significant enrichments for genes involved in mitochondrial function (Table S3). Those transcripts included: *Dlat* (dihydrolipoamide S-acetyltransferase, the E2 compoment of the pyruvate dehydrogenase complex linking glycolysis to TCA cycle), *Cs* (citrate synthase in TCA cycle), and *Cox18* (cytochrome c oxidase assembly protein 18). Three genes in the glycolysis/gluconeogenesis pathway (*Pgk1* (phosphoglycerate kinase 1), *Pgam1* (phosphoglycerate mutase 1) and *Tpi1* (triosephosphate isomerase 1) were also enriched (with uncorrected *p*-value at 0.0036 and Benjamini corrected *p*-value at 0.085, approaching significance). Of those 145 probe sets, 58 (including *Pgk1*, *Pgam1*, *Tpi1*, *Dlat*, *Cs* and *Cox18*) were also rhythmic in adult mouse liver (*BH.Q*<0.1 in GSE11923 [30]; Table S2). In addition, *IRS2* (insulin receptor substrate 2) was also found to be rhythmically expressed in both fetal and adult livers (Table S2).

### Detection of highly fluctuated transcripts by coefficient of variation filtering of fetal liver microarray data

**Figure 3:**
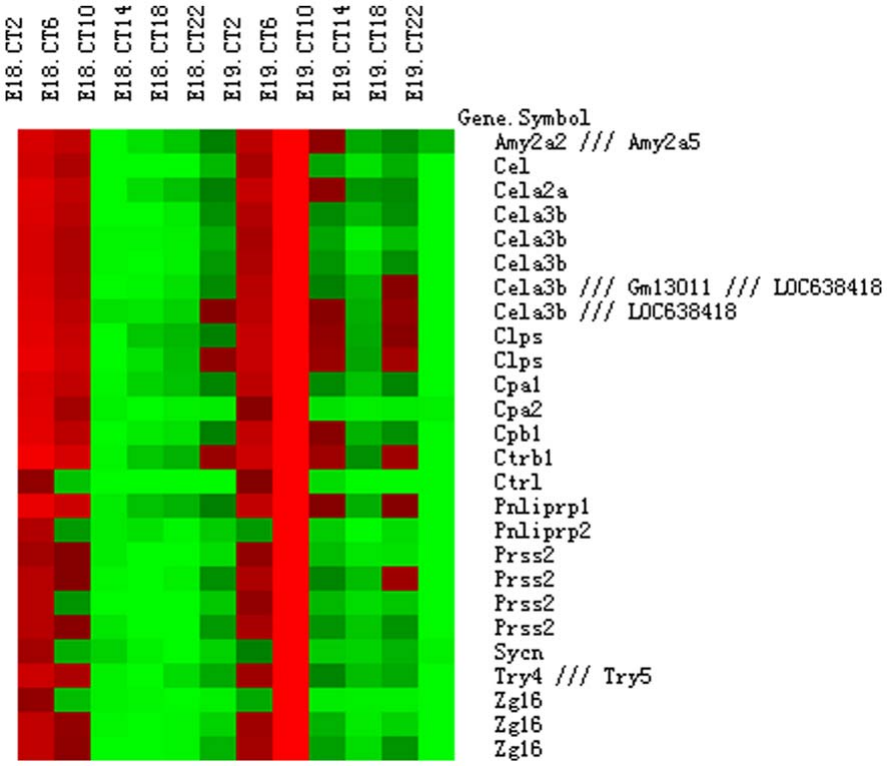
Heatmap of pancreatic exocrine enzyme-related probe sets in series 2 data. Normalized log2 scale expression values for the probe sets representing transcripts related to pancreatic exocrine enzymes and zymogen secretion from series 2 data were plotted. Log2 scale data were used to facilitate viewing of all expression peaks. More dramatic variations in expression peaks in natural scale data led to improper viewing of those peaks on heatmap.

**Figure 4:**
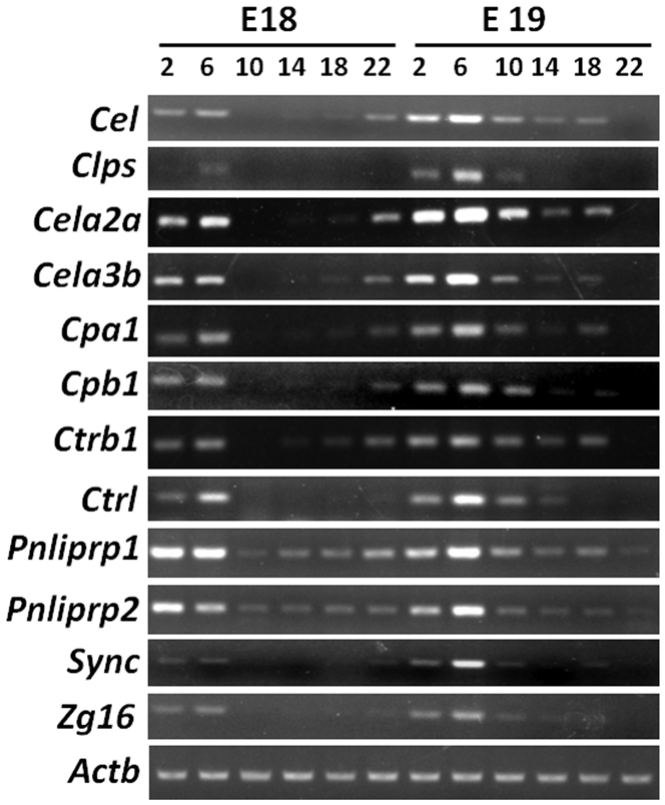
Semi-quantitative RT-PCR analyses of pancreatic exocrine enzymes-related transcripts in fetal liver tissues. Equal amounts of starting RNA from the 12 time points of series 2 fetal liver tissues were reverse transcribed and subject to semi-quantitative PCR. PCR cycles were adjusted depending on transcripts abundance. Products were visualized by agarose gel electrophoresis.

We also observed that the expression values for some transcripts dramatically fluctuated in fetal liver and had high peak to trough ratios (sometimes with fold differences in the hundreds). We performed data filtering (criteria: coefficient of variation ≥0.5) on the expression values from the 12 time points examined on E18 and E19 for all probe sets in our two data series. One-hundred-sixty-six common probe sets (representing 132 unique DAVID IDs) were found between our two series of data (Table S4). Within those highly variable transcripts, 26 showed regular daily expression peaks that aligned together (Figures 3 and S3) on heatmaps, suggesting coordinated transcription. Twenty-two of the probe sets were matched to known pancreatic exocrine enzymes involved in food digestion [31]: *Amy2a* (amylase 2a2/5), *Cela* (chymotrypsin-like elastase 2A and 3B), *Cel* (carboxyl ester lipase), *Clps* (colipase), *Cpa* (carboxypeptidase A1 and A2), *Cpb* (carboxypeptidase B1), *Ctrb1* (chymotrypsinogen B1), *Ctrl* (chymotrypsin-like), *Pnliprp* (pancreatic lipase related protein 1 and 2), *Prss* (protease, serine, 2; also known as *Try2*), and *Try* (trypsin 4/5). Four additional probe sets representing two unique transcripts, *Zg16* (zymogen granule protein 16) and *Sync* (syncollin) were related to zymogen secretion [31], [32]. We performed semi-quantitative RT-PCRs using fetal liver tissues to confirm some of these transcripts. The abundance of PCR products also varied across time points (Figures 4 and S4), with patterns similar to that on the heatmaps. It was known that high sequence similarities existed between some of the transcripts, such as had been shown by alignment between *Try* and *Prss* proteases, and gene duplications between amylases [33]. Sequencing of the PCR products revealed most amplicons represented the desired products. Amplicons for *Try* and *Prss*, however, matched to related sequences, due to close sequence similarities. Amplicons for *Amy2a* also matched to multiple amylases 2a transcripts, due to their close sequence homologies that have resulted from gene duplication events throughout evolution. It was considered likely that transcripts with high sequence similarities were also expressed similarly, but we did not further pursue this complex issue by gene-specific RT-PCRs. Among the pancreatic exocrine enzymes identified here, *Clps* and *Pnliprp2*, had been previously characterized as rhythmically expressed in liver when adult mice were under constant darkness (but not under LD conditions) [34]. We thus took advantage of the available microarray data on circadian liver transcriptome analyses that had been deposited in the public database [10], [30] and searched for those pancreatic exocrine enzymes-related transcripts to determine their expression patterns under various conditions in adult mice. Most of those transcripts showed regular expression spikes, spaced about 21 hours apart, according to an adult mouse liver circadian transcriptome analyses at one-hour resolution (GSE 11923 of [30]; Table S5 and Figure S5). We then performed semi-quantitative RT-PCR using RNAs from adult male mouse livers that were collected under constant darkness (DD). Expression peaks occurred at about CT18 for those transcripts (Figure S6). One previous study had comprehensively addressed the contributions made by the liver clock and feeding on the hepatic transcriptome [10]. Our analyses of the microarray data (GSE13093 in [10] and GSE11923 in [30]) indicated that, in the liver, those pancreatic exocrine enzymes-related transcripts were regulated by feeding patterns regardless of the oscillation status of the liver clock in adult mice (Table S5 and Figure S6; also see Discussion). Moreover, the expression values for those transcripts were significantly rhythmic in our series 2 fetal liver data, but not rhythmic in our series 1 data (by JTK_CYCLE analysis; Table S4). We also observed differences in those transcripts' expression profiles on heatmaps (Figures 3 and S3) and by semi-quantitative RT-PCR, particularly on late E19 (Figures 4 and S4). These differences were not readily explicable, but might be attributable to variations in maternal feeding (see Discussion).

## Discussion

We performed transcriptome analysis on fetal mouse liver tissues during late gestation, a stage at which the oscillation status of the circadian clock at the cellular level has yet to be fully defined. High density oligonucleotide microarray allowed a comprehensive overview of the fetal liver transcriptome. As a result, we obtained several novel findings regarding the oscillation status of the circadian clock and the possible biological cues that affect the fetal liver transcriptome.

Previous real-time RT-PCR studies on developmental expression of clock genes in the fetal rat and mouse livers failed to detect rhythmic expression for several clock genes [19], [21]. We confirmed this observation in our microarray analysis. In addition, we did not detect rhythmic expression patterns for many of the other transcripts that are known to be rhythmically expressed in the adult liver. Expression levels of clock genes and clock-related transcripts also did not change significantly over the two fetal days examined in our two data series, complementing the negative results for rhythmicity detection by JTK_CYCLE analysis. These data supported the conclusion that circadian regulatory cycles are not evident at the tissue level in the fetal mouse liver [21].

We used the newly developed JTK_CYCLE algorithm to detect rhythmicity. This procedure was computationally-efficient and generally outperformed other algorithms in its ability to identify rhythmic transcripts [23]. However, it was not our objective to find and validate the exact number of rhythmic transcripts, as microarray data condensation and rhythmicity detection algorithms all have significant impacts on detection results [30], [35]. In addition, our study dealt with unique tissues that lack rhythmicity for clock genes at the tissue level. We used *p* = 0.1 as the threshold for significance in detection by JTK_CYCLE, without the aid of known rhythmic transcripts. We considered this as a conservative estimation without compromising the true discovery rate. The corrected *p*-values (*BH.Q* in JTK_CYCLE), however, were high for most of the detected rhythmicities. To increase the confidence of rhythmicity detection by JTK_CYCLE in our study, two series of fetal liver tissues were analyzed. These series differed to a certain extent, as the first series was collected from fetal mice with more variable developmental timings. Developmental timing is not expected to significantly affect the expressions of clock genes at late gestation, as tissue level rhythms of clock gene expression were not detected in the fetal liver [21], but it might introduce noise into the data. We anticipated that rhythmic transcripts common between our two data series would represent the truly rhythmic transcripts, regardless of their regulation mechanisms. Those rhythmic transcripts included *Pgk1*, *Pgam1*, *Tpi1*, *Dlat*, *Cs* and *Cox18*, which are involved in glycolysis/gluconeogenesis, TCA cycle and oxidative phosphorylation, key events in cellular metabolism and energy production. Those transcripts might not always be at key regulatory positions within their corresponding pathways, and fetal mitochondria are immature in oxidative metabolism [36]. However, the daily oscillations in those transcripts did suggest circadian changes in metabolism and energy status in fetal liver.

We also found transcripts related to pancreatic exocrine enzymes and zymogen secretion fluctuated in both the fetal and adult livers. Our analyses of GSE11923 and GSE13093 data [10], [30] indicated that feeding patterns had a strong influence on the expression profiles of those pancreatic exocrine enzymes-related transcripts in adult mouse liver (Table S5 and Figure S5). Although daily expression spikes for those transcripts were seen in the livers of adult WT mice under *ad libitum*, restrictive feeding or prolonged fasting conditions, the peaking times clearly differed among those different feeding conditions (Table S5 and Figure S5). When the *Cry1^−/−^ Cry2^−/−^* mice were under *ad libitum* feeding, those transcripts were expressed at basal levels without synchronized fluctuations (Figure S5E), likely due to lack of daily feeding rhythms in the *Cry1^−/−^ Cry2^−/−^* liver under *ad libitum* feeding [10]. However, when those arrhythmic mice were under daily restrictive feeding conditions, expression spikes for those transcripts were found about 7 hours after the feeding window (Figure S5F). Thus, those transcripts were not directly driven by the circadian clock, but their expression profiles could be tuned by the status of the liver clock and systemic cues. It has been shown that in adult mice bearing different mutations in clock genes, those transcripts in the liver differed in their expression profiles relative to the circadian cycle [34]. It is possible that those transcripts are also expressed with different profiles between the maternal and fetal livers, owing to the uncertain status of the fetal liver clock. Unfortunately, in the present study we did not collect maternal liver tissues to test this possibility. Such investigations should be carried out in the future. While only one sharp daily expression peak is typically observed for those transcripts in the adult liver, we observed broader peaks in our two data series and by RT-PCR of fetal liver tissues, suggesting their transcription might be less tightly regulated in the fetal liver. Peaking also differed between our two data series involving late E19, where a prominent peak was observed in series 1 data but less evident in series 2 data. Interestingly, even the rhythmic transcripts that were in common between our two data series also exhibited different phasing patterns (compare Figure 2 and S2). We speculated that the observed discrepancies may be due to differences in the feeding status of pregnant mice at the time of sacrifice. However, restrictive feeding of the dams is required to address this issue in the future.

Overall, our microarray analysis could detect rhythmic transcripts, despite the absence of circadian rhythms of clock gene expression in the fetal liver. We used pooled fetal tissues in the study. It is possible that tissue pooling might have damped the detectable rhythms for clock genes in individual liver. Furthermore, tissue level sampling might have obscured oscillations of different phases among different populations of fetal hepatocytes. Based on our results, it is still difficult to exclude the possibilities that: (1) some fetal hepatocytes (at a relatively small proportion in the fetal liver) possess canonical circadian oscillations, (2) cellular oscillations are weakly coupled in the fetal liver, and (3) unknown clock genes or additional described putative clock genes might also play essential roles in the fetal but not adult liver [37]. Furthermore, other mechanisms distinct from the current clockwork model [4] have been demonstrated as mediators of oscillation processes [38], [39]. Besides those possibilities, systemic cues have been shown as being capable of driving the rhythmic expression of many transcripts in the adult liver and entrain adult liver clock [9], [10]. Fetal liver metabolism relies on maternal nutrient supplies [36], [40], [41]. Fetal liver is capable of sensing maternal nutrient status, at least during maternal fasting [42]. Circadian variations in maternal cues, if present, are likely to affect the fetal liver. In that case, since other rhythmic and fluctuating transcripts were identified in the fetal liver in our study, the lack of detectable circadian rhythms of clock gene expression is peculiar. Peripheral clocks are known to differ in their responsiveness to various cues [12], [43], [44], and also in the developmental onset of circadian oscillations [20]. Whether and how the fetal liver clockwork responds to maternal cues should be examined in future studies.

## Supporting Information

Figure S1 **Heatmap of probe sets representing clock and rhythmic genes in series 1 data.**
(TIF)

Figure S2 **Heatmap of rhythmic probe sets in series 1 data.**
(TIF)

Figure S3 **Heatmap for probe sets representing pancreatic exocrine enzymes-related transcripts in series 1 data.**
(TIF)

Figure S4 **Semi-quantitative RT-PCR analyses of pancreatic exocrine enzymes-related transcripts in series 1 fetal liver.**
(TIF)

Figure S5 **Pancreatic exocrine enzymes-related transcripts' expression in the adult mouse liver under different feeding conditions.** Expression values were calculated for each data series separately, using GC-RMA, and compared. For GSE13093, samples were divided into groups according to genotype and feeding regimen, and then calculated separately using GC-RMA. Groups: 1, GSM327055 and GSM327101–GSM327129; 2, GSM327154–GSM327169; and 3, GSM327130–GSM327153. **A.** Heatmap of transcripts under *ad libtum* feeding in WT mice (GSE11923 data). Peaks occurred at CT25 and CT46. **B.** Heatmap of transcripts under restrictive feeding in WT mice (group 1 of GSE13093). Peak occurred at CT24. **C.** Heatmap of transcripts during fasting in WT mice (group 2 of GSE13093). Peak occurred at CT38. **D.** Heatmap of transcripts during fasting and refed (at CT4) in WT mice (group 2 of GSE13093). Peak occurred at CT8. **E.** Heatmap of transcripts under *ad libtum* feeding in adult *Cry1^−/−^Cry2^−/−^* mice (group 3 of GSE13093). **F.** Heatmap of transcripts under restrictive feeding in *Cry1^−/−^Cry2^−/−^* mice (group 3 GSE13093). Peak occurred at CT40.
(TIF)

Figure S6 **Semi-quantitative PCR analyses of pancreatic exocrine enzymes-related transcripts in adult male mouse liver.** Equal amounts of starting RNA from adult male mouse liver tissues at six time points (four hours resolution across a single DD cycle) were reverse transcribed and subjected to semi-quantitative PCR. Products were visualized by agarose gel electrophoresis. *Pnlip* (pancreatic triglyceride lipase) fluctuated (Table S5 and Figure S5) and peaked at CT18 in the adult liver tissues; however, *Pnlip* was always expressed at basal levels in the fetal liver in our two data series.
(TIF)

Figure S7 **Schedule of fetal tissue collection.** For both series 1 and 2, E0 was defined as the lights-on time after overnight pairing. Dams were released into DD after E16.0 for fetal tissue collection on E18, or after E17.0 for fetal tissue collection on E19. Tissues were collected at four hour intervals of actual time (but designated as circadian time considering the small drift of circadian rhythms that was expected). Livers from adult male mice were also collected under the same schedule.
(TIF)

Table S1 **Results of GC-RMA and JTK_CYCLE analyses of clock genes and known rhythmic genes.**
(XLS)

Table S2 **Rhythmic probe sets and their expression values in the fetal liver.** A total of 145 probe sets from the microarray analysis are presented with their corresponding gene IDs and expression values for the two series of fetal liver data. Of those, 58 probe sets were also identified as found to be also rhythmic (*BH.Q.*<0.1) in the GSE11923 data of adult mice (IDs were also listed).
(XLS)

Table S3 **DAVID annotation and enrichment results of rhythmic transcripts in fetal liver.**
(XLS)

Table S4 **Expression values of pancreatic exocrine enzymes-related probe sets in fetal liver.**
(XLS)

Table S5 **Expression values of pancreatic exocrine enzymes-related probe sets in adult mouse livers.** Adult WT mouse liver data were from GSE11923. Expression values spiked at around CT25 and CT46 for pancreatic exocrine enzymes-related probe sets (corresponding to Figure S5 heatmap). Data in GSE13093 were based on calculations for mice of different genotypes and under different feeding conditions (see Figure S5 legend for details of groupings).
(XLS)

Table S6 **Primers for semi-quantitative and real-time RT-PCR with corresponding GenBank accession numbers.** All PCR amplicons, except where noted, were verified by cloning and sequencing.
(XLS)
