# Supplementary figures and images for: Circadian Rhythms of Fetal Liver Transcription Persist in the Absence of Canonical Circadian Clock Gene Expression Rhythms In Vivo
Source: PLoS One. 2012 Feb 23;7(2):e30781. doi: 10.1371/journal.pone.0030781 (PMC3285613; doi:10.1371/journal.pone.0030781)

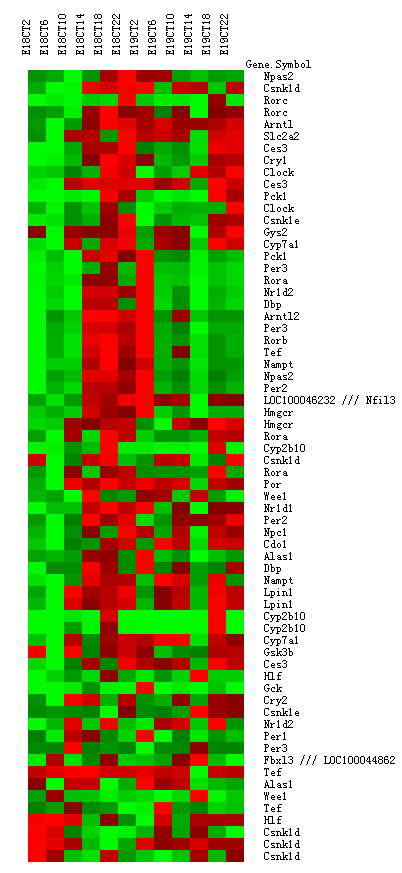

Supplement: Figure S1 — Heatmap of probe sets representing clock and rhythmic genes in series 1 data. (TIF) [file pone.0030781.s001.tif]

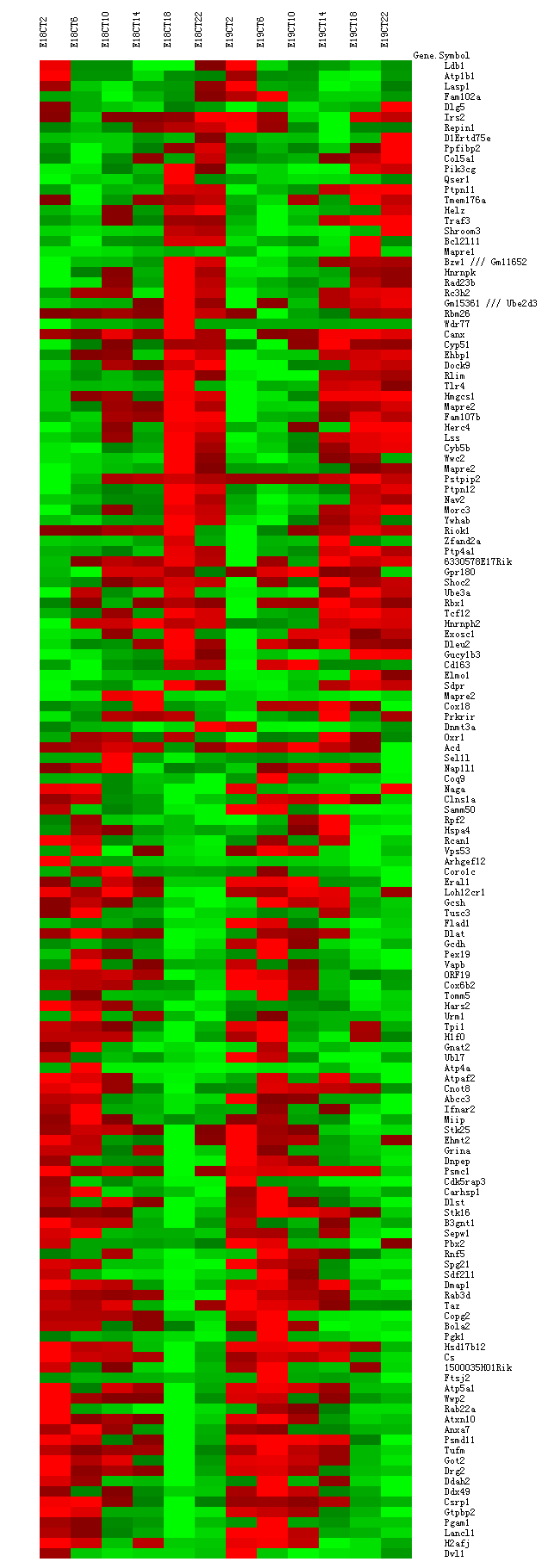

Supplement: Figure S2 — Heatmap of rhythmic probe sets in series 1 data. (TIF) [file pone.0030781.s002.tif]

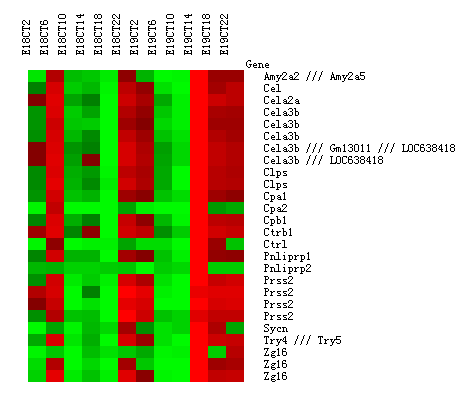

Supplement: Figure S3 — Heatmap for probe sets representing pancreatic exocrine enzymes-related transcripts in series 1 data. (TIF) [file pone.0030781.s003.tif]

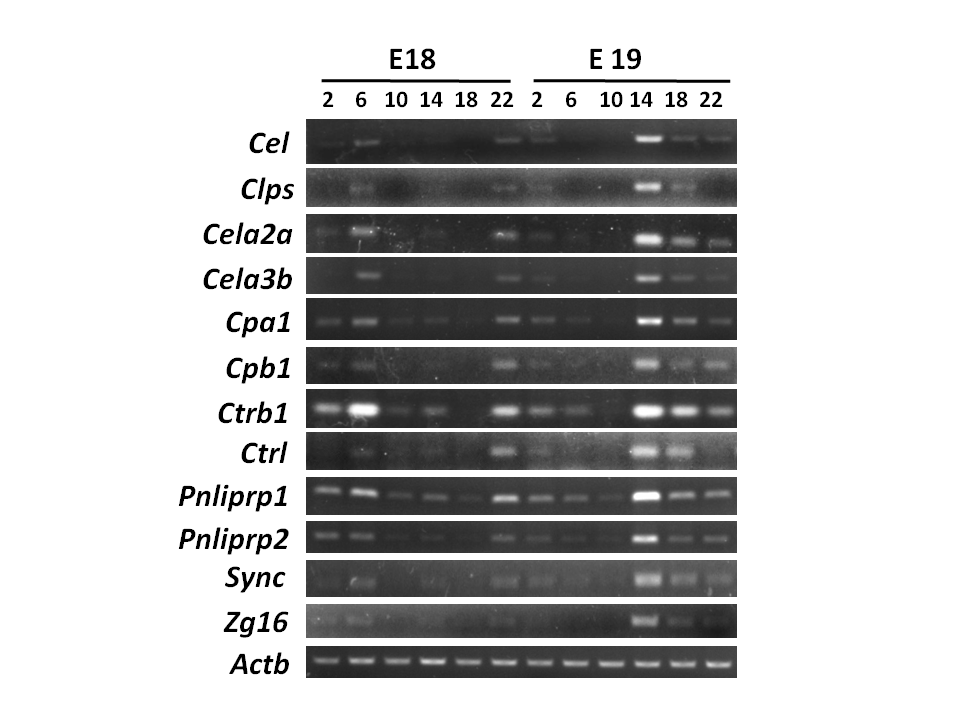

Supplement: Figure S4 — Semi-quantitative RT-PCR analyses of pancreatic exocrine enzymes-related transcripts in series 1 fetal liver. (TIF) [file pone.0030781.s004.tif]

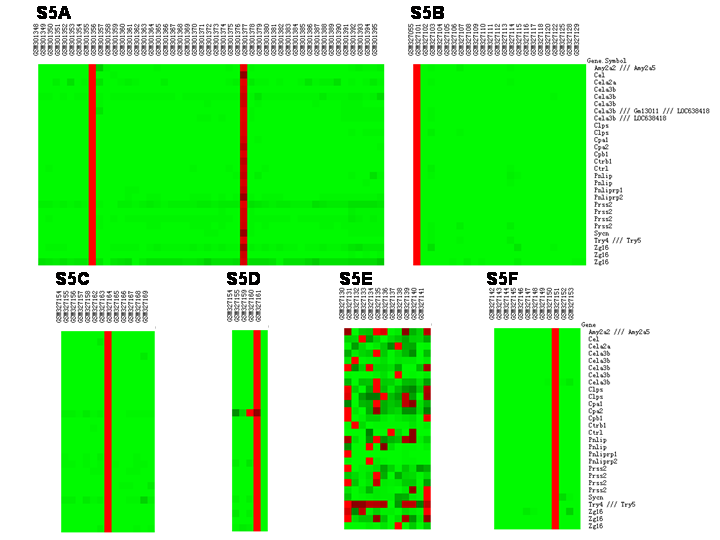

Supplement: Figure S5 — Pancreatic exocrine enzymes-related transcripts' expression in the adult mouse liver under different feeding conditions. Expression values were calculated for each data series separately, using GC-RMA, and compared. For GSE13093, samples were divided into groups according to genotype and feeding regimen, and then calculated separately using GC-RMA. Groups: 1, GSM327055 and GSM327101–GSM327129; 2, GSM327154–GSM327169; and 3, GSM327130–GSM327153. A. Heatmap of transcripts under ad libtum feeding in WT mice (GSE11923 data). Peaks occurred at CT25 and CT46. B. Heatmap of transcripts under restrictive feeding in WT mice (group 1 of GSE13093). Peak occurred at CT24. C. Heatmap of transcripts during fasting in WT mice (group 2 of GSE13093). Peak occurred at CT38. D. Heatmap of transcripts during fasting and refed (at CT4) in WT mice (group 2 of GSE13093). Peak occurred at CT8. E. Heatmap of transcripts under ad libtum feeding in adult Cry1−/−Cry2−/− mice (group 3 of GSE13093). F. Heatmap of transcripts under restrictive feeding in Cry1−/−Cry2−/− mice (group 3 GSE13093). Peak occurred at CT40. (TIF) [file pone.0030781.s005.tif]

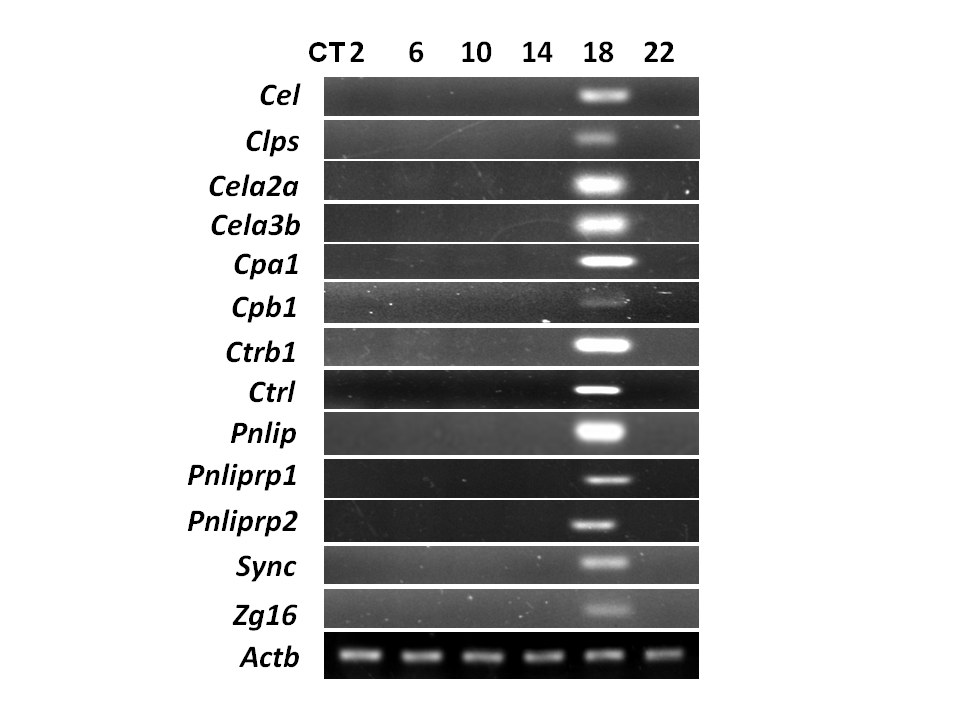

Supplement: Figure S6 — Semi-quantitative PCR analyses of pancreatic exocrine enzymes-related transcripts in adult male mouse liver. Equal amounts of starting RNA from adult male mouse liver tissues at six time points (four hours resolution across a single DD cycle) were reverse transcribed and subjected to semi-quantitative PCR. Products were visualized by agarose gel electrophoresis. Pnlip (pancreatic triglyceride lipase) fluctuated (Table S5 and Figure S5) and peaked at CT18 in the adult liver tissues; however, Pnlip was always expressed at basal levels in the fetal liver in our two data series. (TIF) [file pone.0030781.s006.tif]

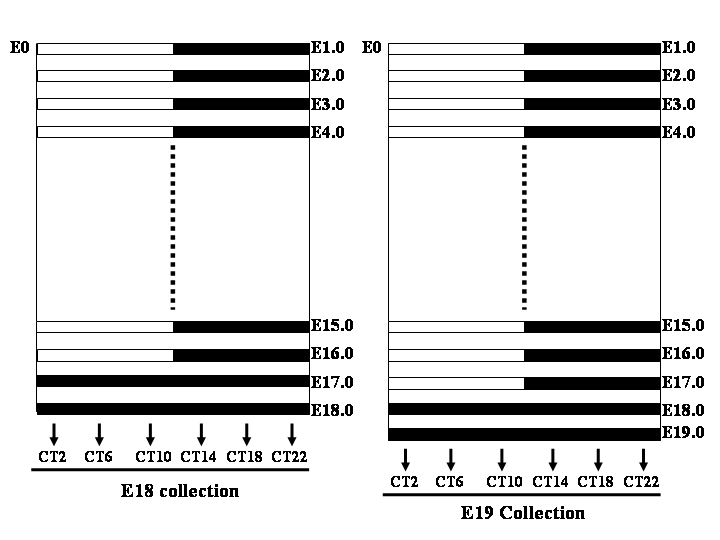

Supplement: Figure S7 — Schedule of fetal tissue collection. For both series 1 and 2, E0 was defined as the lights-on time after overnight pairing. Dams were released into DD after E16.0 for fetal tissue collection on E18, or after E17.0 for fetal tissue collection on E19. Tissues were collected at four hour intervals of actual time (but designated as circadian time considering the small drift of circadian rhythms that was expected). Livers from adult male mice were also collected under the same schedule. (TIF) [file pone.0030781.s007.tif]
